# The importance of selection at the level of the pair over 25 years in a natural population of birds

**DOI:** 10.1002/ece3.835

**Published:** 2013-10-22

**Authors:** Mats Björklund, Lars Gustafsson

**Affiliations:** Department of Animal Ecology, Evolutionary Biology Centre, Uppsala UniversityUppsala, Sweden

**Keywords:** collared flycatchers, long-term data, model selection, pair-level selection

## Abstract

Knowledge of the pattern of selection in natural populations is fundamental for our understanding of the evolutionary process. Selection at higher levels has gained considerable theoretical support in recent years, and one possible level of selection is the breeding pair where fitness is a function of the pair and cannot be reduced to single individuals. We analyzed the importance of pair-level selection over 25 years in a natural population of the collared flycatcher. Pair-level selection was significant in five and probably in another 9 years. The relative importance of pair-level selection varied over years and can have stronger or the same strength as directional selection. This means that selection can act on the combination of the breeding pair in addition to selection on each individual separately. Overall, the conservative estimates obtained here show that this is a potentially important form of selection.

## Introduction

Natural selection is the covariance between phenotypes and fitness. This means that selection can operate only if there is variation among phenotypes and the fitness of the phenotypes. It has in recent years been increasingly clear that the definition of selection is very general and not necessarily restricted to single individuals, even though this might be the most common case (e.g., Okasha 2006; Frank 2012; Okasha and Paternotte 2012). Thus, this definition has been extended to groups of any size, such that if there is a group phenotype and group fitness, then selection can act among groups at a strength determined by the covariance between the group phenotype and the group fitness. In addition, there can be selection among individuals within a group that might act in the same or in the opposite direction as the selection among groups. Even if the notion of group selection has a long troublesome history and has been regarded with (often well-supported) skepticism in the scientific community, the theoretical foundation of multilevel selection is now so strong that the relative importance of this form of selection is now merely an empirical question (Okasha 2006; Bijma and Wade 2008).

The mathematical and analytical details of multilevel selection usually come in two forms (Okasha 2006; the Price equation (e.g., Frank 2012) or through contextual analysis (Heisler and Damuth 1987). However, these ways of analysis both consist of two parts, selection between groups and selection among individuals within groups, the main difference between the approaches resides in the interpretation of the individual and group terms (Okasha and Paternotte 2012). If there is no variation in fitness among individuals within a group, then the only possible selection that can act is between groups provided that there is variation between groups. This can, for example, be groups where the competition between individuals is repressed, or in clones. However, in the cases where we have selection among groups but no, or only minor, individual selection within groups, then among-group selection might be more common than previously thought. One such case is the reproduction of strictly monogamous pairs, where there is no variation in reproductive fitness between the pair members, which means that all variation in reproductive fitness will be found among pairs. Hence, there are strong reasons to analyze the potential impact of selection at the level of the pair, as this is in many cases the level where most variation in fitness resides, and hence have the largest opportunity for selection.

The reproductive output of a pair is a combination of the male and female phenotype. This means that when we analyze selection acting at reproduction, we need to incorporate both male and female phenotypes as they both affect the reproductive output and cannot be reduced to single individuals. This does not differ from traditional analysis of selection on individual phenotypes where selection on a given trait is estimated controlling for the selection on other correlated traits, as well as selection on trait combinations. The difference is that we need to treat the pair as the phenotype and analyze selection on the different traits and trait combinations in the traditional way (e.g., Lande and Arnold 1983; Arnold and Wade 1984). Thus, we estimate the selection acting on male traits holding female traits constant, just the way we estimate selection on each trait holding the other traits constant. The main difference is that we now include interaction terms with male and female traits. The interaction terms are clearly properties of the pair and cannot be reduced to single individuals. In this article, we will call this pair-level selection as the selection estimated is a property of the pair. There are no *a priori* reasons to believe pair-level interactions to be absent; on the contrary, there has long been known that the combination of old and young members of a pair can affect reproductive output (e.g., Coulson 1966). Thus, there are no reasons to assume this is restricted to age alone, but can appear for any trait combination (e.g., Robinson et al. 2012).

Although it is clear that pair-level selection can be important in theory, the issue is whether it is common in natural populations or common enough to be a factor to take into account in future analyses of selection. Here, we aim to estimate the importance of pair-level selection using a data set collected on the collared flycatcher on the island of Gotland over 25 years (1981–2005; e.g., Gustafsson and Qvarnström 2006). This species is basically monogamous with a certain but small proportion of polygynous matings within a year (9%; Qvarnström et al. 2003; Gustafsson and Qvarnström 2006) and some extra-pair parentage (15% of offspring in 33% of the broods; Sheldon and Ellegren 1999). As we have data on EPC for only 2 years, we are not able to include this information in the analysis. However, this has probably a very small effect on the analysis (Data S2). The species has extensive biparental care, and it has been shown that the number of recruits from a two parents attending is almost four times larger than for single females (Gustafsson and Qvarnström 2006). Thus, the reproductive output is a function of the combined effort of the two phenotypes, and there are therefore reasons to expect that pair-level selection is acting. To test this, we contrast four models, two with only one sex included, one model with both males and females included but with no interactions, and one model where interaction terms between male and female phenotypes are included. Thus, we are not searching for the globally best model as the number of possible models to compare will be overwhelming. Moreover, there are theoretical arguments not to do so (c.f. Burnham and Anderson 2002).

## Material and Methods

Data on reproductive success and phenotypic measures were collected using standardized methods as part of an ongoing (1980-) long-term study of a wild collared flycatcher population breeding on the island of Gotland, Sweden (57°30′N, 18°33′E; e.g., Qvarnström et al. 2006). In general, the pairs were a new combination of males and females every year; of the 5276 pairs used in this study, we only found 17 (0.3%) cases with the same male and female breeding in two subsequent years. Most males were only found to breed once (66.0%), 20.6% were found to breed twice, and hence, 13.4% of the males bred more than twice. The corresponding figures for females where 69.5% bred once, 20.0% bred twice, and thus, 10.5% of the females were found breeding more than twice. Thus, even if some individuals were found breeding more than in 1 year, there was no pair fidelity between years. Adults and nestlings were individually banded. Birds born in 1 year did not breed that year, but returned from their African wintering sites the year after to breed. Thus, the youngest breeders were 1 year old and that constituted the major group of breeders (males 32.5%, females 39.3%). A small proportion of males mate polygynously (about 9–14% per year; Gustafsson and Qvarnström 2006). As the identification of polygynous males is uncertain (secondary females are often abandoned), only males that were known to be monogamous are included in this analysis.

We analyzed selection on adult male and female age and male and female wing length (ref) in each of the 25 years. Age was also chosen as it is known to be important for breeding success in this species (Gustafsson and Pärt 1990) and is probably related to experience. Wing length is strongly correlated with overall size in both males and females (unpublished), and in some years, there is assortative mating with regard to wing length (see below). We also used hatching date in the model, which is known to strongly influence fitness in this species (Lindén et al. 1992). This is a property of the brood and hence the pair, and in order to compare total selection intensities between the different models, we excluded any selection on hatching in the comparisons as it could not be attributed to one sex only. We used the standard techniques for analyzing multivariate selection (e.g., Lande and Arnold 1983) treating either a single sex or the pair as the unit of selection. Thus, we have a linear relationship between a trait and fitness (directional selection), a quadratic relationship (stabilizing/disruptive selection), and a relationship between trait combinations and fitness (correlational selection; for details see Data S1). As a measure of fitness, we used the number of recruits, that is,, the number of breeding offspring the following year, for each pair, transformed into relative fitness by dividing with mean fitness. Note that the estimated quadratic selection coefficient needs to be multiplied with two (Stinchcombe et al. 2008). As we treat the pair as the unit of selection, we simply combine males and females as shown in Supplementary Material 1 such that we get estimates on, for example, male wing length taking into account correlations with male age, female wing length, and female age. To incorporate pair-level selection, here defined as the selection on the phenotype of one sex that is dependent on the phenotype of the other sex, we added an interaction term between male and female traits (see Data S1), in addition to the correlational selection between traits within a sex.. This is a property of the pair, and hence, a significant selection coefficient is evidence for selection at the level of the pair, that is, selection on a combination of male and female traits.

All traits were standardized to unit variance and a mean of zero each year. As the number of recruits is Poisson-distributed, we used a generalized linear model (Poisson log-model) rather than a classic multiple regression to test for significance (Shaw and Geyer 2010) and chose the model with the lowest AIC (Akaike Information Criterion). We only used selection coefficients that had 95% intervals that differed from zero. The estimates of selection coefficients are not accurate using this approach, even though the statistical testing is so (Shaw and Geyer 2010). In reality, however, the estimates from the traditional approach and from the generalized model were strongly correlated, which means that the estimates are close to published data from other taxa, and we therefore use the estimates from the generalized linear model. We used four models; one with males only, one with females only, one with both males and females but with no interaction terms, and finally, a pair-level model where also the interactions between the phenotypes were included. The details of the different models are given in Data S1. We compared the models using the Akaike Information Criterion (AIC), or more precisely the AICc, which is a correction for finite sample sizes defined as AICc = AIC + [2*k*(*k* + 1)]/(*n-k*-1), where *k* is the number of parameters in the model and *n* is sample size (Burnham and Anderson 2002). To display the difference between the models, we calculated the relative likelihood defined as exp((AICc_min_−AICc_*i*_)/2), where AICc_min_ is the lowest AICc of the models compared and AICc_i_ is the AICc of model *i*. The relative likelihood gives the probability that the alternative model is in fact the best model. Thus, if the relative likelihood is 0.05, then there is a 5% probability that the alternative model is in fact better. We used this level as a criterion of significance in the comparison of the models. When two models were not significantly different, we used model averaging to get the estimates of the selection coefficients.

We decided to use only two traits known to be important to fitness and analyzed the interaction between those. This does not mean that other traits or other trait combinations are not important. In contrast, we assume that the interactions are at the level of the whole phenotype with an unknown number of traits. However, the choice of using only two traits (and hatching date) is based on tractability of the models in relation to sample size. With the traits used, the most complex model had 15 parameters to be estimated, and adding one trait, for example tarsus length, would result in a full model with an additional 12 parameters, that is, 27 in total. This would require very large sample sizes to get good estimates with reasonable standard errors.

To assess the suitability of our model(s), we used the methods derived by Moorad and Wade (2013) that estimates the proportion of the variance in relative fitness that can be accounted for by the model, in standard statistical language *R*^2^. Their approach allows decomposition of total *R*^2^ into components of directional, quadratic, and correlational selection. We estimate total *R*^2^ as well as *R*^2^ for directional, quadratic, and pair-level (male and female interaction components) selection.

We tested the temporal correlation between mean temperature in April and mean fitness, opportunity for selection, and total selection. We used data from a weather station run by the Swedish Meteorological and Hydrological Institute situated about 10 km from the main nest-box areas. To account for any temporal autocorrelation and trends, we prefiltered the mean temperature in April before calculating the cross-correlation function (cf. Chatfield 2004, pp. 158–159). The expected value is then 0, and the variance is 1/N. Thus, values outside ± 2/√N are significant at the 5% level (Chatfield 2004).

## Results

**Figure 1:**
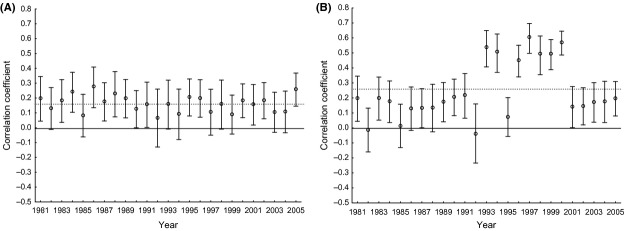
Assortative mating over the period 1981–2005. Bars denote 95% intervals. (A) age, (B) wing length

We found significant assortative mating in age in 14 of the 25 years and in wing length in 20 of the 25 years. The assortative mating with regard to male and female age was more or less constant over the year, averaging 0.16 (test of homogeneity χ^2^ = 19.46, *df* = 24, *P* = 0.39; Fig. 1A). When we controlled for the fact that age and wing length were often correlated, we found significant assortative mating in wing length in 16 of the 25 years. The assortative mating with regard to wing length varied significantly over the years (mean = 0.25, test of homogeneity χ^2^ = 209.2, *df* = 24, *P* ≪ 0.001; Fig. 1B), with a few years in the 1990s where the strength of assortative mating considerably stronger than in all other years. If these are removed, the strength of assortative mating is not significantly heterogeneous and average 0.14 (χ^2^ = 17.64, *df* = 13, *P* = 0.25).

**Figure 2:**
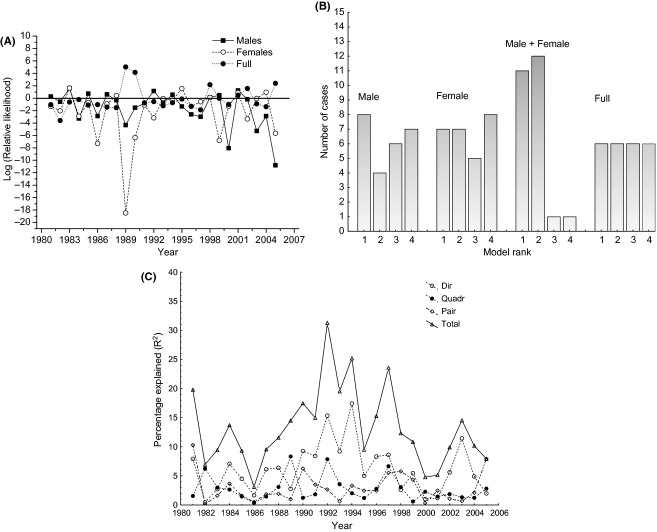
Results from the comparison of the four models. (A) Comparison of the different models in terms of (Log) relative likelihood. All comparisons are in relation to the male+female model. (B) Frequency distribution of model ranks where 1 denotes the best model. (C) The proportion of variance in relative fitness explained by different modes of selection

When comparing the four models, we found that the model that incorporated both male and female phenotypes was the best, or second best model, in 22 of 25 years (Fig. 2A, B). The next best model was males only (best or next best 12 times), followed by females only (11 times), and the full model (11 times). However, in five cases was the full model the one with the lowest AICc, and in these cases was the difference to the alternative models large (Fig. 2A, Table S1). The relative likelihood of the full model was high (>0.05) in another 9 years (Table S1). The single-sex models, being the best ones in some years, were clearly inferior in other years such as in 1989 where the full model was the by far the best model (Fig. 2A; Table S1), and the female only model had a relative likelihood that was substantially lower than the alternative models.

**Figure 3:**
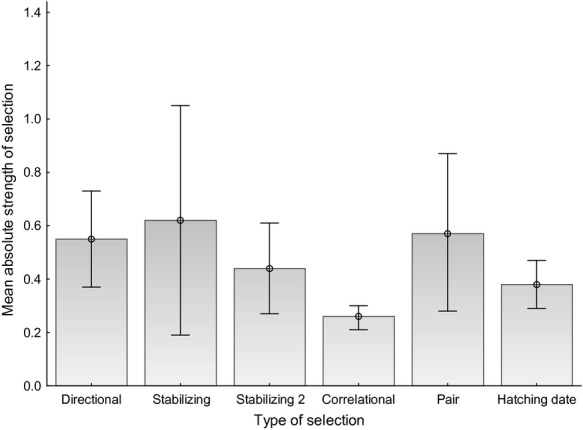
Mean absolute strength of selection. The second bar of stabilizing selection is when year 2005 is excluded (see text for details). Bars denote 95% intervals.

Directional selection was the most common form of selection, followed by quadratic and correlational and pair-level selection being equally common (Fig 3). There were no patterns in the sign of selection with positive and negative selection coefficients being about equally common. The only exception from this is selection on hatching date, which was predominantly negative; this indicates that selection for earlier hatching date was common. However, the magnitude of selection varied between years from being zero in some years to being strong in other years (Table S2).

The proportion of variance in relative fitness (*R*^2^) that could be accounted for by our model(s) ranged from 3.1% (1986) up to 31.3% (1992), with a mean of 13.2%. Directional selection accounted for on average 6.2% (range 0.5–17.4%), quadratic selection on average 2.8% (range 0.4–8.3%), and pair selection on average 3.0% (range 0.2–10.3%). The difference between directional and the other two types of selection was significant (*P* = 0.0023, Wilcoxon test), but the difference between quadratic and pair selection was not (*P* = 0.78, Wilcoxon test).

**Figure 4:**
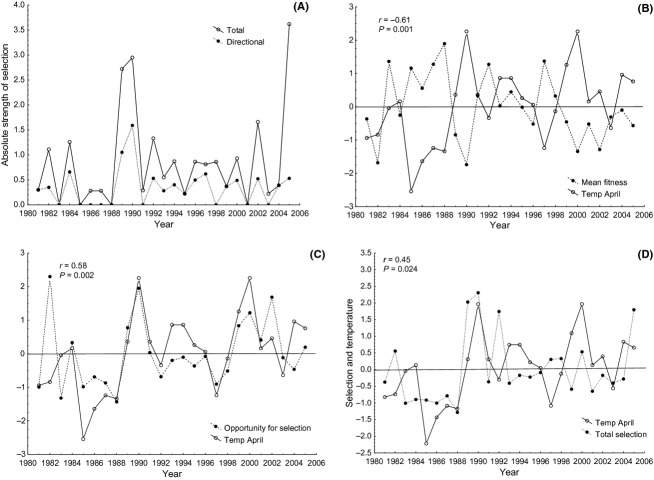
(A) The absolute strength of total and directional selection (sexes combined) over the period 1981–2005. (B) Mean fitness and mean temperature in April over the period 1981–2005. (C) Opportunity for selection and mean temperature in April over the period 1981–2005. (D) Total selection and mean temperature in April over the period 1981–2005. In figures B-D, values are standardized to zero mean and unit variance to allow easy comparison

The mean strength of selection (using significant selection coefficients only) was about equal for directional, quadratic, and pair-level selection, with correlational selection being significantly weaker than directional selection (*U* = 78.5, *P* = 0.041, U-test) and quadratic selection (*U* = 38, *P* = 0.020, U-test), but not different from pair-level selection (*P* > 0.5, *U*-test; Fig. 4). The strength of quadratic selection was very strong in 2005, which makes the standard error large. If we remove this year, the pattern is still same. Pair-level selection was not significantly less in strength compared with directional selection (*P* > 0.45, *U*-test).

The absolute strength of selection varied considerably over time (Fig. 4A). To get an understanding of the causes of this varying selection, we looked at mean fitness in relation to mean temperature in April and found a significant negative correlation (Fig. 4B); mean fitness was low when mean temperature was high and vice versa. The variance in fitness, the opportunity for selection, was positively correlated with mean temperature (Fig. 4C). Hence, a warm early spring increased the variance among pairs in the number of recruits. As the opportunity for selection only gives the scope for selection, we also compared the total absolute selection and temperature and we found a significant positive correlation (Fig. 4D).

**Figure 5:**
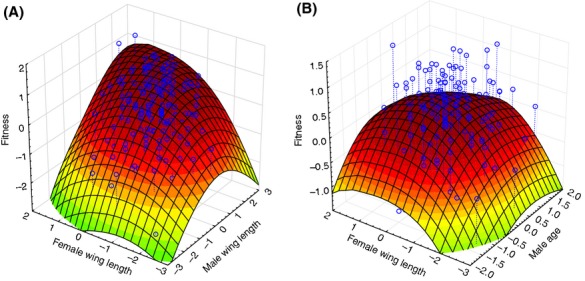
An example of social selection relating to male and female wing length in the year 1990. (B) Same for female wing length and male age.

Most of the significant pair-level selection coefficients were between age and wing length (7 times), between male and female wing length once, and between male and female age three times (Table S2). To illustrate this form of selection, we picked two cases, between male and female wing length in 1990 (Fig. 5A), where fitness increases with male wing length but more so when the wing length of the partner was large. The same was true for female wing length. Hence, pairs where both parents had longer wings had a higher fitness than other combinations. Another example is between female wing length and male age in 1990, where there seems to be an optimum around mean female wing length and male age (Fig. 5B).

## Discussion

We found that assortative mating was common over 25 years in this population of flycatchers for the two traits analyzed. In most years, the model incorporating selection at the level of the pair was superior to the single-sex models. This shows that when analyzing selection acting on one sex, the selection in the other sex needs to be taken into account. We have also shown that, in some years, models incorporating interaction terms between the sexes, here called pair-level selection, were far better than simpler models. This happened in 5 years, but the full model was not significantly worse in another 9 years. The number of cases of pair selection was slightly lower than the total number of cases with significant directional selection, but at the same level as the number of significant cases of quadratic selection for each trait and sex. The strength of pair-level selection was at the same level as the strength of other forms of selection when it occurred. In total, our model accounted for a substantial proportion of the variance in relative fitness in some years. Taken together, this shows that the variance in reproductive output of a pair is most often a combination of male and female phenotypes, and in some years, at least, a result of the interaction of male and female phenotypes, that is, pair-level selection. The results strongly indicate that this form of selection is more common than previously thought and needs to be considered in future studies of selection. Treating the pair as the unit of selection, rather than the separate sexes thus gives an additional insight into the selection process.

In one year (1990), the pair-level selection was in terms of a positive selection on male and female wing length. This suggests that the selection is acting on overall size of the pairs; in other words, pairs where both parents were large had a higher fitness than other combinations of body sizes of the pair members. In 2 years (1998, 2005), we found that older pairs were, on average, more successful than other combinations of age. Given that age has repeatedly been shown to be an important factor for breeding success in this and other species of birds (e.g., Coulson 1966; Perrins and McCleery 1985; Gustafsson and Pärt 1990), it is somewhat surprising that this was not found more often. The mean difference in age between the sexes is 1 year, with a small variance (1.17), suggesting that in most years, there is not enough variation for selection to act with regard to age of the pair members. We find positive selection for a combination of male wing and female age in 2 years (1989, 2002). This means that pairs with large males and older females had, on average, a higher relative fitness than other combinations. This suggests that in these years, size and experience were selected for but only in a certain combination and not overall. The most common form of pair-level selection was on female wing length and male age, being positive in 1 year (1988) and negative in 4 years (1990, 1998, 2002, 2005). Thus, in 1 year, we have selection for large females and older males, thus selection for size and experience, but in different way compared with 1989 and 2002. The selection surface for 1990 is shown Figure 5B and suggests that there is an intermediate combination of female wing length (size) and male age that have the highest relative fitness. In other words, the fitness benefit of large females is dependent on the age of the male and vice versa. All these show that the exact form of the pair-level selection is not predictable and can change between years, all dependent on the ecological conditions in the particular year.

We included male and female age in the analysis and found significant effects of age on fitness, either separately or in combination. The concept of age is complicated as there cannot be selection on age itself, but rather on something that age is a proxy for. One such thing would be arrival time, such that older birds are able to obtain a better territory. We have no information on arrival times of males and females, but if we use laying date as a proxy for arrival time, we find that older females lay their eggs on average 2 days (95% interval 1–3 days) before first-year-old females. This difference is small, and even if the mean values differ, the overlap in laying date is about 95% for young and old females. This suggests that this is not the most important factor. Instead, we suggest that age is a proxy for experience, that is, in some years, experienced birds have a higher fitness, and in some years, only if they are mated to a partner with the same experience or larger size.

We also found that the strength of selection was related to a possible cause of variation in fitness, namely mean temperature in April. Warmer years lead to lower mean fitness, a higher variance in relative fitness, hence a larger opportunity for selection, and also stronger selection. The strong relationship between the opportunity for selection and the actual strength of selection is interesting, as opportunity for selection only puts an upper limit for the selection that can occur (Arnold and Wade 1984), rather than being the selection itself. A possible causal link between temperature and selection intensities can be the peak of insect abundance with a mismatch of the peak of breeding and peak of insect abundance (e.g., Stenseth and Mysterud 2002; te Marvelde et al. 2011). In an early year, the peak of the food abundance is earlier than the peak of the food demand, and hence, the mean number of recruits, which is our estimate of fitness, decreases. We will also like to point out that selection can also be acting on other levels, as the reproductive output of a given pair might as well be influenced by competition with other pairs and species.

The mean temperature differed greatly between years and hence so did direction and strength of selection (contra Morrissey and Hadfield 2011), and we have a factor that correlate with these fluctuations in selection. It is worth stressing that in a system like this, with a strong dependence on insect abundance, which itself is strongly depending on the weather conditions during breeding, this is exactly what we would expect. The variation among years in onset of spring and the conditions during breeding (temperature, rain, extreme events) varies greatly, and in this study, the date of the mean first egg has a range over years of about 14 days. Thus, as breeding conditions change so much between years, we can expect selection to vary accordingly.

The model choice procedure we used is based on parsimony, and thus, complex models are down-weighted in favor of simpler ones. Yet, in 6 years, the most complex model including interactions between male and female phenotypes had the lowest AIC. Thus, using only two traits (+ hatching date) and a model selection procedure that penalizes complex models, we are still able to find strong support for the action of pair-level selection in this population. Consequently, there are good reasons to believe that pair-level selection is important in this population, and we see no reasons to believe that this is not the case also in other populations, only severely underestimated.

The approach taken is to some extent related to the recent development of social selection theory (e.g., Wolf et al. 1999; McGlothlin et al. 2010; Westneat 2011), pushing an idea that is not new (e.g., Griffing 1967, 1981; West-Eberhard 1979, 1983) but up to recent years has been largely forgotten. In this framework, the fitness of an individual is not only influenced by its own phenotype but the phenotype of other individuals with which the individual interact. The social selection coefficient is then the selection arising from the social interactions. What we have shown here is that the fitness of the pair is to a varying extent dependent on the combination of the phenotypes of a breeding pair. The difference between our study, studies of monogamous pairs in general, and the study of social selection is that in our case, fitness cannot be partitioned to single individuals as the unit that has a fitness is the breeding pair. The two approaches are similar in the sense that to get a more complete description of the selection that is acting in natural population interactions between individuals needs to be taken into account in much more detail than has been carried out before. While there are a number of studies that have demonstrated social selection (for some recent examples see Formica et al. 2011; Procter et al. 2012), we are not aware of any study using the approach taken here. The results we got, however, strongly suggest that studies of selection at the level of the pair can be rewarding and add to a deeper understanding of the selection process.

In this species, as in many other mainly monogamous birds, there is a certain proportion of extra-pair mating. This means that the fitness of males and females is not exactly the same, as the male might not be the father of all the offspring in the clutch, and in addition might have sired offspring in other clutches. This creates a within-pair variation in fitness, which in turn affects the total selection and in the end response to selection. However, there might still be selection on combinations of the phenotypes of the pair, here called pair-level selection, as this is a property of the pair that cannot be reduced to a lower level. We have, unfortunately, no data on the extent of extra-pair mating for every year of study, but only for 2 years. Using these data (see Data S2), we show that the opportunity for within-pair selection is about 6% of the opportunity of selection between pairs. Thus, even if within-pair selection cannot be disregarded, it is likely to be of less important in most cases compared to selection between pairs.

This way of analyzing is by no means restricted to monogamous pairs. In cases where one sex mates have multiple partners, we can equally well use the breeding group as the unit of selection, but with the addition of possible selection within each groups. In these polygamous cases, there can be variation in fitness within a sex, and this variation can be related to the combination of traits of this sex, but also on trait combinations with the other sex. This adds complexity to the model, but the logic is the same. Our point is that the analysis of selection might be as affected by including trait combinations with other individuals in all cases in the same way as in our study. If the aim is to understand patterns of selection in nature and its causes, then this form of selection needs to be taken into account.

## Supporting Information

**Data S1.** Description of the analysis of selection

**Data S2.** The influence of extra-pair copulations on the opportunity for selection

**Table S1.** Results and comparison of models for each year

**Table S2.** Selection coefficients and their standard errors for each trait and year. We only include coefficients that were estimated in the best model (see Table S1). *ne* stands for not estimated, while *ns* stands for nonsignificant.
