# Supplementary material for: The importance of selection at the level of the pair over 25 years in a natural population of birds
Source: Ecol Evol. 2013 Oct 22;3(13):4610–9. doi: 10.1002/ece3.835 (PMC3856758; doi:10.1002/ece3.835)
Supplement: Supplementary file 1 [file ece30003-4610-SD1.docx]

Data S1. A description of the selection models used in the analysis. Age^2^ and wing^2^ denotes quadratic selection on age and wing, respectively.

Summary of the models

| Model | Parameters included | No parameters |
| --- | --- | --- |
| Male | Hatching date, age, wing, age^2^, wing^2^, age x wing | 6 |
| Female | Hatching date, age, wing, age^2^, wing^2^, age x wing | 6 |
| Pair | Male, Female | 11 |
| Full | Male, Female, male x female age, male x female wing, male age x female wing, male wing x female age | 15 |

*One-sex models*

To estimate the selection gradients, β, we used the standard way of regressing relative fitness on standardized trait values

β = P^-1^s

where P is the phenotypic variance-covariance matrix (cf Lande & Arnold 1983). To make the following treatment easier to follow we can expand this for trait 1 and trait 2 as

$$\left[ \begin{matrix} \beta_{1} \\ \beta_{2} \end{matrix} \right]=\left[ \begin{matrix} \sigma_{11} & \sigma_{12} \\ \sigma_{21} & \sigma_{22} \end{matrix} \right]\left[ \begin{matrix} s_{1} \\ s_{2} \end{matrix} \right]$$

Where σ_11_ and σ_22_ are the phenotypic variances of traits 1 and 2, respectively, and σ_12_ is the phenotypic covariance between traits 1 and 2.

We also estimated the quadratic selection coefficients (stabilizing and disruptive selection) using the squared trait values, and the cross-correlations (correlational selection) as

$$\gamma=\left[ \begin{matrix} \gamma_{11} & \gamma_{12} \\ \gamma_{21} & \gamma_{22} \end{matrix} \right]$$

Where γ_11_ and γ_22_ is the quadratic coefficients for trait 1 and 2, respectively, and γ_12_ is the correlational selection between traits 1 and 2.

*Two-sex model*

In this model we combined males and females to account for correlations between male and female traits (assortative mating)

$$\left[ \begin{matrix} \beta_{m1} \\ \begin{matrix} \begin{matrix} \beta_{m2} \\ \beta_{f2} \end{matrix} \\ \beta_{f2} \end{matrix} \end{matrix} \right]=\left[ \begin{matrix} \begin{matrix} \sigma_{m11} \\ \sigma_{m21} \end{matrix} & \begin{matrix} \begin{matrix} \sigma_{m21} & \begin{matrix} \sigma_{f11} & \sigma_{m1f2} \end{matrix} \end{matrix} \\ \begin{matrix} \sigma_{m22} & \begin{matrix} \sigma_{m2f1} & \sigma_{m2f2} \end{matrix} \end{matrix} \end{matrix} \\ \begin{matrix} \sigma_{m1f1} \\ \sigma_{m1f2} \end{matrix} & \begin{matrix} \begin{matrix} \sigma_{m2f1} & \begin{matrix} \sigma_{f11} & \sigma_{f12} \end{matrix} \end{matrix} \\ \begin{matrix} \sigma_{m2f2} & \begin{matrix} \sigma_{f21} & \sigma_{f22} \end{matrix} \end{matrix} \end{matrix} \end{matrix} \right]\left[ \begin{matrix} s_{m1} \\ \begin{matrix} s_{m2} \\ \begin{matrix} s_{f1} \\ s_{f2} \end{matrix} \end{matrix} \end{matrix} \right]$$

whereσ_m1f1_ is the phenotypic correlation between male trait 1 and female trait 1, etc.

The quadratic coefficients in this model was then estimated as

$$\gamma=\left[ \begin{matrix} \begin{matrix} \gamma_{m11} \\ \end{matrix} & \begin{matrix} \begin{matrix} & \begin{matrix} & \end{matrix} \end{matrix} \\ \begin{matrix} \gamma_{m22} & \begin{matrix} & \end{matrix} \end{matrix} \end{matrix} \\ \begin{matrix} \\ \end{matrix} & \begin{matrix} \begin{matrix} & \begin{matrix} \gamma_{f11} & \end{matrix} \end{matrix} \\ \begin{matrix} & \begin{matrix} & \gamma_{f22} \end{matrix} \end{matrix} \end{matrix} \end{matrix} \right]$$

i.e. we only estimated the stabilizing/disruptive gradients.

*Full model*

The difference between the two-sex model and the full model is that we in the full model also estimated the correlated selection of male and female traits. This is a property of the pair and hence these are the selection coefficients that give information about selection at the level of the pair. Hence, we used the matrix

$$\gamma=\left[ \begin{matrix} \begin{matrix} \gamma_{m11} \\ \gamma_{m21} \end{matrix} & \begin{matrix} \begin{matrix} \gamma_{m12} & \begin{matrix} \gamma_{m1f1} & \gamma_{m1f2} \end{matrix} \end{matrix} \\ \begin{matrix} \gamma_{m22} & \begin{matrix} \gamma_{m2f1} & \gamma_{m2f2} \end{matrix} \end{matrix} \end{matrix} \\ \begin{matrix} \gamma_{m1f1} \\ \gamma_{m1f2} \end{matrix} & \begin{matrix} \begin{matrix} \gamma_{m2f1} & \begin{matrix} \gamma_{f11} & \gamma_{f12} \end{matrix} \end{matrix} \\ \begin{matrix} \gamma_{m2f2} & \begin{matrix} \gamma_{f21} & \gamma_{f22} \end{matrix} \end{matrix} \end{matrix} \end{matrix} \right]$$
