# Supplementary material for: The importance of selection at the level of the pair over 25 years in a natural population of birds
Source: Ecol Evol. 2013 Oct 22;3(13):4610–9. doi: 10.1002/ece3.835 (PMC3856758; doi:10.1002/ece3.835)
Supplement: Supplementary file 2 [file ece30003-4610-SD2.docx]

Table S1. Results of the analysis of selection. The table show the sample size (N), number of parameters estimated (k), raw AIC, AICc, difference in AICc between the best and the alternative models (∆) and relative likelihood, for each year of study. Note that the relative likelihood (last column) is scaled by the model with the lowest AIC. Thus, the best model have a relative likelihood of 1.0.

| Year | Model | N | k | AIC | AICc | ∆ | Rel. Lik. |
| --- | --- | --- | --- | --- | --- | --- | --- |
| 1981 | Male | 162 | 6 | 387.49 | 388.03 | 0.00 | 1 |
|  | Female | 162 | 6 | 394.83 | 395.37 | 7.34 | 0.025 |
|  | M+F | 162 | 11 | 387.18 | 388.94 | 0.91 | 0.64 |
|  | Full | 162 | 15 | 388.15 | 391.44 | 3.41 | 0.18 |
|  |  |  |  |  |  |  |  |
| 1982 | Male | 189 | 6 | 630.43 | 630.89 | 2.89 | 0.24 |
|  | Female | 189 | 6 | 637.22 | 637.68 | 9.68 | 0.0089 |
|  | M+F | 189 | 11 | 626.51 | 628.00 | 0.00 | 1.00 |
|  | Full | 189 | 15 | 641.68 | 644.45 | 16.45 | 0.00027 |
|  |  |  |  |  |  |  |  |
| 1983 | Male | 178 | 6 | 425.17 | 425.66 | 0.10 | 0.95 |
|  | Female | 178 | 6 | 425.07 | 425.56 | 0.00 | 1.00 |
|  | M+F | 178 | 11 | 431.20 | 432.79 | 7.23 | 0.027 |
|  | Full | 178 | 15 | 432.58 | 435.54 | 9.98 | 0.0068 |
|  |  |  |  |  |  |  |  |
| 1984 | Male | 199 | 6 | 489.42 | 489.87 | 15.31 | 0.00047 |
|  | Female | 199 | 6 | 487.83 | 488.27 | 13.71 | 0.011 |
|  | M+F | 199 | 11 | 473.14 | 474.56 | 0.00 | 1.00 |
|  | Full | 199 | 15 | 472.85 | 475.48 | 0.92 | 0.63 |
|  |  |  |  |  |  |  |  |
| 1985 | Male | 195 | 6 | 470.81 | 471.26 | 0.00 | 1.00 |
|  | Female | 195 | 6 | 477.46 | 477.91 | 6.65 | 0.036 |
|  | M+F | 195 | 11 | 472.98 | 474.43 | 3.17 | 0.20 |
|  | Full | 195 | 15 | 476.80 | 479.48 | 8.23 | 0.016 |
|  |  |  |  |  |  |  |  |
| 1986 | Male | 210 | 6 | 512.52 | 512.94 | 13.46 | 0.0012 |
|  | Female | 210 | 6 | 532.90 | 533.32 | 33.84 | < 10^-6^ |
|  | M+F | 210 | 11 | 498.14 | 499.48 | 0.00 | 1.00 |
|  | Full | 210 | 15 | 501.72 | 504.20 | 4.72 | 0.094 |
|  |  |  |  |  |  |  |  |
| 1987 | Male | 253 | 6 | 595.68 | 596.02 | 0.00 | 1.00 |
|  | Female | 253 | 6 | 602.70 | 603.04 | 7.02 | 0.030 |
|  | M+F | 253 | 11 | 597.54 | 598.64 | 2.62 | 0.27 |
|  | Full | 253 | 15 | 603.21 | 605.24 | 9.21 | 0.010 |
|  |  |  |  |  |  |  |  |
| 1988 | Male | 164 | 6 | 384.98 | 385.52 | 3.48 | 0.18 |
|  | Female | 164 | 6 | 381.50 | 382.04 | 0.00 | 1.00 |
|  | M+F | 164 | 11 | 381.89 | 383.63 | 0.45 | 0.45 |
|  | Full | 164 | 15 | 387.33 | 390.57 | 8.53 | 0.014 |
|  |  |  |  |  |  |  |  |
| 1989 | Male | 265 | 6 | 600.39 | 600.72 | 43.36 | < 10^-6^ |
|  | Female | 265 | 6 | 665.50 | 665.83 | 108.47 | < 10^-6^ |
|  | M+F | 265 | 11 | 579.52 | 580.57 | 23.20 | < 10^-6^ |
|  | Full | 265 | 15 | 555.44 | 557.37 | 0.00 | 1.00 |
|  |  |  |  |  |  |  |  |
| 1990 | Male | 244 | 6 | 737.18 | 737.53 | 26.55 | < 10^-6^ |
|  | Female | 244 | 6 | 759.30 | 759.65 | 48.67 | < 10^-6^ |
|  | M+F | 244 | 11 | 729.09 | 730.23 | 19.24 | 0.0001 |
|  | Full | 244 | 15 | 708.88 | 710.99 | 0.00 | 1.00 |
|  |  |  |  |  |  |  |  |
| 1991 | Male | 167 | 6 | 440.505 | 441.03 | 4.17 | 0.12 |
|  | Female | 167 | 6 | 441.738 | 442.26 | 5.40 | 0.07 |
|  | M+F | 167 | 11 | 435.160 | 436.86 | 0.00 | 1.00 |
|  | Full | 167 | 15 | 437.024 | 440.20 | 3.34 | 0.19 |
|  |  |  |  |  |  |  |  |
| 1992 | Male | 103 | 6 | 226.066 | 226.94 | 0.00 | 1.00 |
|  | Female | 103 | 6 | 245.994 | 246.87 | 19.93 | 0.00005 |
|  | M+F | 103 | 11 | 228.808 | 231.71 | 4.77 | 0.092 |
|  | Full | 103 | 15 | 228.479 | 234.00 | 7.06 | 0.029 |
|  |  |  |  |  |  |  |  |
| 1993 | Male | 146 | 6 | 329.794 | 330.40 | 3.74 | 0.15 |
|  | Female | 146 | 6 | 326.681 | 327.29 | 0.83 | 0.73 |
|  | M+F | 146 | 11 | 324.690 | 326.66 | 0.00 | 1.00 |
|  | Full | 146 | 15 | 328.464 | 332.16 | 5.50 | 0.064 |
|  |  |  |  |  |  |  |  |
| 1994 | Male | 137 | 6 | 324.747 | 325.39 | 0.00 | 1.00 |
|  | Female | 137 | 6 | 328.727 | 329.37 | 3.98 | 0.14 |
|  | M+F | 137 | 11 | 325.436 | 327.55 | 2.15 | 0.34 |
|  | Full | 137 | 15 | 326.814 | 330.78 | 5.39 | 0.068 |
|  |  |  |  |  |  |  |  |
| 1995 | Male | 324 | 6 | 818.665 | 818.93 | 13.25 | 0.0013 |
|  | Female | 324 | 6 | 805.414 | 805.68 | 0.00 | 1.00 |
|  | M+F | 324 | 11 | 811.752 | 812.60 | 6.92 | 0.031 |
|  | Full | 324 | 15 | 811.578 | 813.14 | 7.46 | 0.024 |
|  |  |  |  |  |  |  |  |
| 1996 | Male | 233 | 6 | 624.51 | 624.89 | 12.19 | 0.0023 |
|  | Female | 233 | 6 | 618.60 | 618.98 | 6.28 | 0.043 |
|  | M+F | 233 | 11 | 611.50 | 612.70 | 0.00 | 1.00 |
|  | Full | 233 | 15 | 616.55 | 618.77 | 6.06 | 0.048 |
|  |  |  |  |  |  |  |  |
| 1997 | Male | 170 | 6 | 381.62 | 382.14 | 14.06 | 0.00088 |
|  | Female | 170 | 6 | 370.53 | 371.05 | 2.98 | 0.23 |
|  | M+F | 170 | 11 | 366.40 | 368.07 | 0.00 | 1.00 |
|  | Full | 170 | 15 | 373.44 | 376.56 | 8.49 | 0.014 |
|  |  |  |  |  |  |  |  |
| 1998 | Male | 188 | 6 | 359.72 | 360.19 | 9.85 | 0.0073 |
|  | Female | 188 | 6 | 359.71 | 360.18 | 9.84 | 0.0073 |
|  | M+F | 188 | 11 | 358.96 | 360.47 | 10.13 | 0.0063 |
|  | Full | 188 | 15 | 347.54 | 350.34 | 0.00 | 1.00 |
|  |  |  |  |  |  |  |  |
| 1999 | Male | 232 | 6 | 713.95 | 714.32 | 0.00 | 1.00 |
|  | Female | 232 | 6 | 747.54 | 747.92 | 33.59 | < 10^-6^ |
|  | M+F | 232 | 11 | 715.17 | 716.37 | 2.04 | 0.36 |
|  | Full | 232 | 15 | 714.15 | 716.37 | 2.05 | 0.36 |
|  |  |  |  |  |  |  |  |
| 2000 | Male | 304 | 6 | 879.21 | 879.50 | 37.25 | < 10^-6^ |
|  | Female | 304 | 6 | 847.99 | 848.28 | 6.03 | 0.049 |
|  | M+F | 304 | 11 | 841.34 | 842.25 | 0.00 | 1.00 |
|  | Full | 304 | 15 | 845.14 | 846.81 | 4.56 | 0.10 |
|  |  |  |  |  |  |  |  |
| 2001 | Male | 210 | 6 | 658.37 | 658.79 | 0.00 | 1.00 |
|  | Female | 210 | 6 | 659.96 | 660.37 | 1.58 | 0.45 |
|  | M+F | 210 | 11 | 662.96 | 664.29 | 5.50 | 0.064 |
|  | Full | 210 | 15 | 659.47 | 661.95 | 3.15 | 0.21 |
|  |  |  |  |  |  |  |  |
| 2002 | Male | 262 | 6 | 773.80 | 774.14 | 8.26 | 0.016 |
|  | Female | 262 | 6 | 788.39 | 788.72 | 22.84 | 0.00001 |
|  | M+F | 262 | 11 | 772.07 | 773.13 | 7.25 | 0.027 |
|  | Full | 262 | 15 | 763.92 | 765.88 | 0.00 | 1.00 |
|  |  |  |  |  |  |  |  |
| 2003 | Male | 232 | 6 | 518.13 | 518.50 | 24.55 | < 10^-6^ |
|  | Female | 232 | 6 | 494.02 | 494.40 | 0.44 | 0.80 |
|  | M+F | 232 | 11 | 492.75 | 493.96 | 1.00 | 1.00 |
|  | Full | 232 | 15 | 495.87 | 498.09 | 4.13 | 0.13 |
|  |  |  |  |  |  |  |  |
| 2004 | Male | 199 | 6 | 485.98 | 486.42 | 17.71 | 0.00014 |
|  | Female | 199 | 6 | 468.28 | 468.72 | 0.00 | 1.00 |
|  | M+F | 199 | 11 | 471.41 | 472.83 | 4.11 | 0.13 |
|  | Full | 199 | 15 | 476.43 | 479.06 | 10.34 | 0.0057 |
|  |  |  |  |  |  |  |  |
| 2005 | Male | 333 | 6 | 918.76 | 919.03 | 60.97 | < 10^-6^ |
|  | Female | 333 | 6 | 895.09 | 895.35 | 37.30 | < 10^-6^ |
|  | M+F | 333 | 11 | 868.32 | 869.14 | 11.09 | 0.0039 |
|  | Full | 333 | 15 | 856.54 | 858.06 | 0.00 | 1.00 |
