# Supplementary material for: The importance of selection at the level of the pair over 25 years in a natural population of birds
Source: Ecol Evol. 2013 Oct 22;3(13):4610–9. doi: 10.1002/ece3.835 (PMC3856758; doi:10.1002/ece3.835)
Supplement: Supplementary file 3 [file ece30003-4610-SD3.docx]

Data S2. An analysis of the relative importance of extra-pair mating in this population.

There is a certain proportion of extra-pair paternity in this population (Sheldon and Ellegren 1999). This means that, strictly speaking, male and female fitness is not the same over all pair, but there is a variation even within pairs. This can affect the estimation of the level of selection among pairs, depending on the model of analysis chosen. However, since we do not have data from each year on the details of extra-pair paternities we have to rely in indirect methods (simulations) to assess the possible impact, namely through the opportunity of selection, defined as the variance in relative fitness (Arnold and Wade 1984). This puts an upper limit to selection, which means that if the variance among pairs is many times larger than the variance within pairs, selection could be many times larger although this is not necessarily the case. We used the data in Brommer et al. (2010) where the number of extra-pair offspring within each clutch was on average 0.5. The mean number of recruits was 0.55 and in 96 % of the clutches the number of eggs was between 5 and 7 (5: 13.7 %, 6: 57.0 %, 7: 25.3 %). We randomly created 10 000 clutches with clutch sizes in the same proportions as observed. Since the number of recruits is not significantly related to clutch size (at least not in this segment), the number of recruits was drawn from a Poisson distribution with a mean 0.55. We then calculated the mean number of recruits and used this to calculate the opportunity of selection for the pair (= female) component. To simulate extra-pair paternities we randomly assigned the number of extra-pair chicks from a Poisson-distribution with a mean of 0.5. Each male also had a probability of siring a certain number of offspring in another clutch, each with a certain probability of being a recruit as above. This number was added to the males to get an estimate of the variance in male fitness. We then created recruits as above and calculated the variance of relative fitness within each pair, i.e. the opportunity for selection within the pair. Based on these simulations we found that the opportunity of selection between pairs was about 18 times larger than the opportunity of selection within pairs. This suggests that the within-pair selection is indeed negligible compared to the selection among pairs, even though it cannot be ruled out. In the closely related pied flycatcher it has been found that polygynous males suffer more from potential extra-pair matings than monogamous males (ratio = 1.6), while the possibilities to get an extra-pair copulation was the same (Alatalo et al. 1987). The mating system in the collared flycatcher is the same as in the pied flycatcher, which means that the mean number of extra-pair copulations is likely to be lower if only monogamous males are included as in our study.
