# Supplementary material for: The importance of selection at the level of the pair over 25 years in a natural population of birds
Source: Ecol Evol. 2013 Oct 22;3(13):4610–9. doi: 10.1002/ece3.835 (PMC3856758; doi:10.1002/ece3.835)
Supplement: Supplementary file 4 [file ece30003-4610-SD4.docx]

| Year | HDate | MW | MW2 | MA | MA2 | MAW | FW | FW2 | FA | FA2 | FAW | W | A | MAFW | MWFA |
| --- | --- | --- | --- | --- | --- | --- | --- | --- | --- | --- | --- | --- | --- | --- | --- |
| 1981 | ns | ns | ns | ns | ns | ns |  |  |  |  |  |  |  |  |  |
| 1982 | 0.15  (0.077) | -0.35  (0.11) | ns | ns | ns | 0.29  (0.073) | ns | -0.24  (0.089) | ns | ns | ns |  |  |  |  |
| 1983 |  |  |  |  |  |  | ns | ns | ns | ns | ns |  |  |  |  |
| 1984 | -0.50 (0.11) | ns | ns | -0.24  (0.12) | ns | 0.38  (0.14) | 0.18  (0.080) | ns | ns | ns | ns |  |  |  |  |
| 1985 | -0.20  (0.076) | ns | ns | ns | ns | ns |  |  |  |  |  |  |  |  |  |
| 1986 | ns | ns | ns | ns | ns | ns | ns | ns | ns | ns | -0.28  (0.11) |  |  |  |  |
| 1987 | -0.28  (0.072) | ns | -0.14  (0.061) | ns | ns | ns |  |  |  |  |  |  |  |  |  |
| 1988 | -0.31  (0.11) | ns | ns | ns | ns | ns | ns | ns | ns | ns |  |  |  |  |  |
| 1989 | -0.31  (0.090) | ns | 0.23  (0.057) | 0.46  (0.18) | ns | -0.30  (0.12) | -0.59  (0.24) | -0.17  (0.085) | ns | ns | ns | ns | ns | 0.42  (0.11) | ns |
| 1990 | -0.44  (0.092) | 0.80  (0.15) | -0.21  (0.086) | -0.47  (0.14) | ns | ns | ns | ns | 0.32  (0.13) | ns | ns | 0.42  (0.13) | ns | -0.52  (0.12) | ns |
| 1991 | -0.39  (0.10) | ns | 0.15  (0.068) | ns | ns | ns | ns | ns | ns | ns | ns |  |  |  |  |
| 1992 | -0.76  (0.17) | -0.52  (0.16) | -0.40  (0.13) | ns | ns | ns |  |  |  |  |  |  |  |  |  |
| 1993 | -0.40  (0.11) |  |  |  |  |  | ns | ns | ns | ns | 0.25  (0.12) |  |  |  |  |
| 1994 | -0.81  (0.12) | ns | ns | -0.40  (0.13) | 0.26  (0.065) | -0.21  (0.088) |  |  |  |  |  |  |  |  |  |
| 1995 | -0.25  (0.056) |  |  |  |  |  | ns | ns | -0.22  (0.088) | ns | ns |  |  |  |  |
| 1996 | -0.30  (0.079) | -0.20  (0.087) | ns | ns | 0.11  (0.053) | 0.16  (0.063) | 0.30  (0.10) | ns | ns | ns | Ns |  |  |  |  |
| 1997 | ns | -0.24  (0.12) | ns | ns | ns | ns | 0.38  (0.18) | ns | ns | ns | -0.19  (0.084) |  |  |  |  |
| 1998 | ns | ns | ns | ns | ns | ns | ns | ns | ns | ns | ns | ns | 0.44  (0.14) | -0.43  (0.19) | ns |
| 1999 | -0.29  (0.082) | 0.37  (0.092) | ns | ns | ns | ns |  |  |  |  |  |  |  |  |  |
| 2000 | ns | 0.17  (0.079) | ns | ns | ns | ns | ns | 0.11  (0.038) | 0.32  (0.10) | ns | 0.22  (0.058) |  |  |  |  |
| 2001 | ns |  |  |  |  |  | ns | ns | ns | ns | ns |  |  |  |  |
| 2002 | -0.34  (0.077) | -0.24  (0.01) | -0.16  (0.077) | ns | -0.18  (0.074) | ns | 0.28  (0.089) | ns | ns | ns | ns | ns | ns | -0.19  (0.093) | 0.28  (0.089) |
| 2003 | -0.58  (0.088) | ns | ns | ns | ns | ns | ns | ns | ns | ns | -0.24  (0.10) |  |  |  |  |
| 2004 | -0.24  (0.088) |  |  |  |  |  | ns | ns | 0.39  (0.15) | ns | ns |  |  |  |  |
| 2005 | -0.26  (0.19) | ns | ns | -0.53  (0.14) | -0.49  (0.17) | 0.29  (0.086) | ns | 0.12  (0.054) | ns | -0.61  (0.16) | ns | ns | 0.18  (0.086) | -0.20  (0.072) | ns |
